# The complete chloroplast genome sequence of *Hippophae rhamnoides* subsp. *sinensis*

**DOI:** 10.1080/23802359.2020.1719932

**Published:** 2020-02-03

**Authors:** Songfeng Diao, Guoyun Zhang, Caiyun He, Aiguo Duan, Jianguo Zhang

**Affiliations:** a State Key Laboratory of Tree Genetics and Breeding & Key Laboratory of Tree Breeding and Cultivation, National Forestry and Grassland Administration, Research Institute of Forestry, Chinese Academy of Forestry, Beijing, China;; b Non-timber Forestry Research and Development Center, Chinese Academy of Forestry, Key Laboratory of Non-timber Forest Germplasm Enhancement & Utilization of National Forestry and Grassland Administration, Zhengzhou, China;; c Collaborative Innovation Center of Sustainable Forestry in Southern China, Nanjing Forestry University, Nanjing, China

**Keywords:** *Hippophae rahmnoides* subsp. *sinensis*, chloroplast genome, phylogenetic analysis, genetic information

## Abstract

The complete chloroplast genome sequence of *Hippophae rahmnoides* subsp. *sinensis* was characterized from Illumina pair-end sequencing. The chloroplast genome of *H. rahmnoides* subsp. *sinensis* was 156,355 bp in length, containing a large single-copy region (LSC) of 84,002 bp, a small single-copy region (SSC) of 19,055 bp, and two inverted repeat (IR) regions of 26,649 bp. The overall GC content is 36.6%, while the corresponding values of the LSC, SSC, and IR regions are 64.5%, 69.2%, and 60.1%, respectively. The genome contains 131 complete genes, including 88 protein-coding genes, 38 tRNA genes (29 tRNA species), and 8 rRNA genes (4 rRNA species). The neighbour-joining phylogenetic analysis showed that *H. rahmnoides* subsp. *sinensis* and *H. rahmnoides* clustered together as sisters to other *H. rahmnoides* species.

## Introduction

*Hippophae rahmnoides* subsp. *sinensis* is the most widely distributed subspecies of *Hippophae* Linn. in China (Jia et al. 2012). It not only has the characteristics of drought tolerance, cold resistance, salt and alkali resistance, and barren resistance, but also has the ecological effects of wind and sand fixation, water and soil conservation, and soil improvement (He et al. 2015). Its fruit is rich in vitamin, protein, amino acid, unsaturated fatty acid, flavonoid, a variety of trace elements and other 428 kinds of beneficial nutrients and bioactive substances, with lipid-lowering, anti-inflammatory, anti-cancer and treatment of coronary heart disease, gastrointestinal diseases, liver and kidney protection and other significant effects (Zhang et al. 2019) and enjoys the reputation of ‘green gold’ (Jimenez-Garcia et al. 2013).

The fresh leaves of *H. rahmnoides subsp. sinensis* were collected from Fengning County, Hebei province, P. R. China (41°14′37″N, 116°38′59″E). Fresh leaves were silica-dried and taken to the laboratory until DNA extraction. The voucher specimen (ZGSJ001) was laid in the Herbarium of Research Institute of Forestry and the extracted DNA was stored in the -80 °C refrigerator of the State Key Laboratory of Tree Genetics and Breeding, Key Laboratory of Silviculture of the State Forestry Administration. We extracted total genomic DNA from 25 mg silica-gel-dried leaf using a modified CTAB method (Doyle 1987). The whole-genome sequencing was then conducted by Biodata Biotechnologies Inc. (Hefei, China) with Illumina Hiseq platform. The Illumina HiSeq 2000 platform (Illumina, San Diego, CA) was used to perform the genome sequence. We used the software MITObim 1.8 (Hahn et al. 2013) and metaSPAdes (Nurk et al. 2017) to assemble chloroplast genomes. We used *H. rahmnoides* (GenBank: NC_035548) as a reference genome. We annotated the chloroplast genome with the software DOGMA (Wyman et al. 2004), and then corrected the results using Geneious 8.0.2 (Campos et al. 2016) and Sequin 15.50 (http://www.ncbi.nlm.nih.gov/Sequin/).

The complete chloroplast genome of *H. rahmnoides* subsp. *sinensis* (GenBank accession number MN857418) was 156,355 bp in length, containing a large single-copy region (LSC) of 84,002 bp, a small single-copy region (SSC) of 19,055 bp, and two inverted repeat (IR) regions of 26,649 bp. The overall GC content is 36.6%, while the corresponding values of the LSC, SSC, and IR regions are 64.5%, 69.2%, and 60.1%, respectively. The genome contains 131 complete genes, including 88 protein-coding genes, 38 tRNA genes (29 tRNA species) and 8 rRNA genes (4 rRNA species).

**Figure 1.**
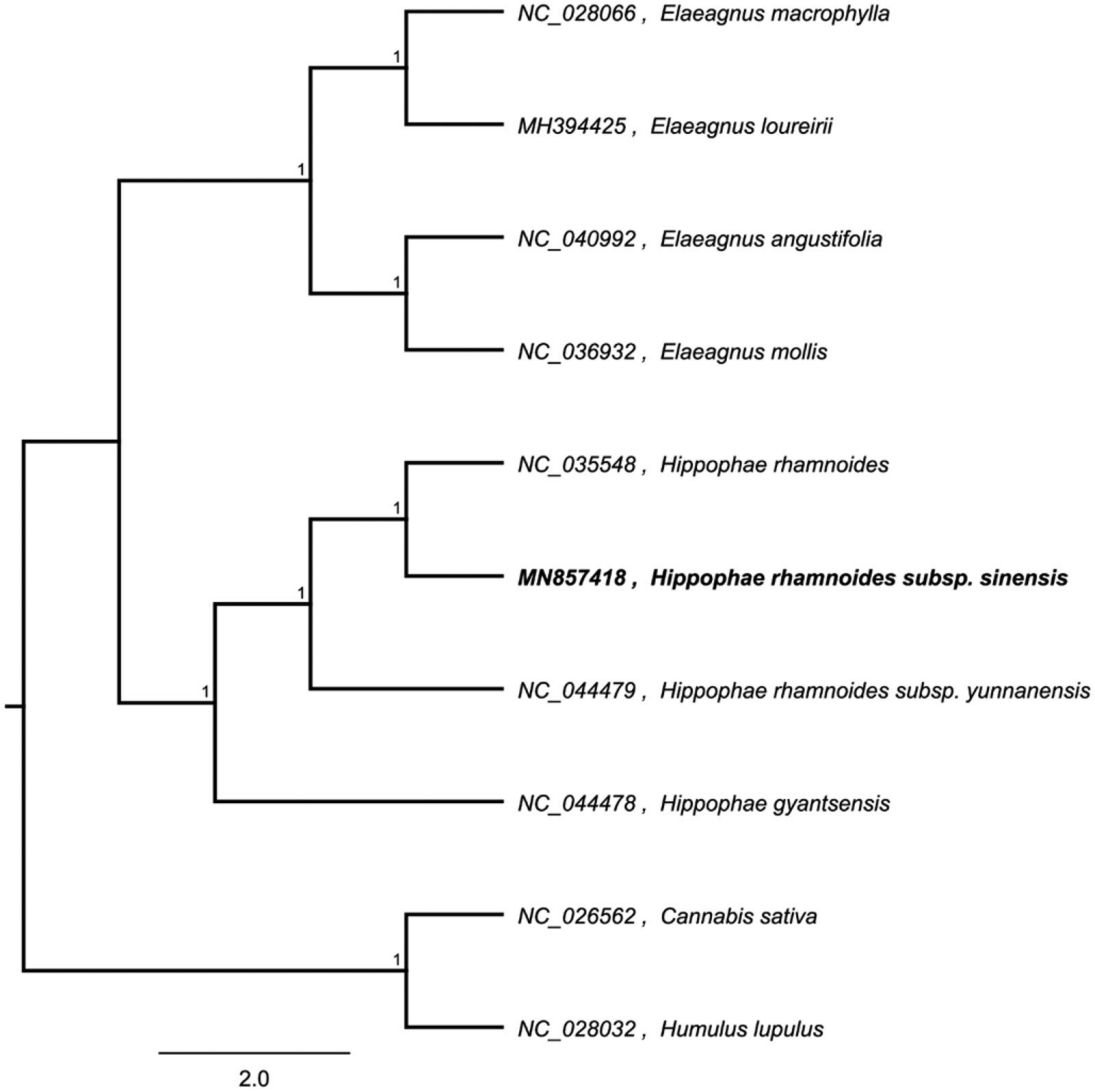
Neighbour-joining (NJ) analysis of *H. rahmnoides* subsp. *sinensis* and other related species based on the complete chloroplast genome sequence.

We used the complete chloroplast genomes sequence of *H. rahmnoides* subsp. *sinensis* and other related species of Elaeagnaceae, *Cannabis sativa* and *Humulus lupulus* as outgroup to construct phylogenetic tree. The 10 chloroplast genome sequences were aligned with MAFFT (Katoh and Standley 2013), and then the Neighbour-joining tree was constructed by MEGA 7.0 (Kumar et al. 2016). The results confirmed that *H. rahmnoides* subsp. *sinensis* and *Hippophae rahmnoides* clustered together as sisters to other *H. rahmnoides* species (Figure 1).
